# Healthcare professionals’ knowledge of the systematic ABCDE approach: a cross-sectional study

**DOI:** 10.1186/s12873-022-00753-y

**Published:** 2022-12-12

**Authors:** Nino H. C. Schoeber, Marjolein Linders, Mathijs Binkhorst, Willem-Pieter De Boode, Jos M. T. Draaisma, Marlies Morsink, Anneliese Nusmeier, Martijn Pas, Christine van Riessen, Nigel M. Turner, Rutger Verhage, Cornelia R. M. G. Fluit, Marije Hogeveen

**Affiliations:** 1 Department of Neonatology, Radboud University Medical Center, Amalia Children’s Hospital, Nijmegen, the Netherlands; 2 Department of Emergency Medicine, Radboud University Medical Center, Nijmegen, the Netherlands; 3 Department of Paediatrics, Radboud University Medical Center Amalia Children’s Hospital, Nijmegen, the Netherlands; 4 Paediatric Intensive Care Unit, Radboud University Medical Center, Nijmegen, the Netherlands; 5 Department of Anaesthesia and Pain, Radboud University Medical Center, Nijmegen, the Netherlands; 6 Division of Vital Functions, University Medical Centre Utrecht, Utrecht, the Netherlands; 7 Intensive Care Unit, Radboud University Medical Center, Nijmegen, the Netherlands; 8 Radboudumc Health Academy, Radboud University Medical Center, Nijmegen, the Netherlands

**Keywords:** ABCDE, Critical care, Life support care, Knowledge, Assessment

## Abstract

**Background:**

The Airway, Breathing, Circulation, Disability and Exposure (ABCDE) approach is a universal, priority-based approach for the assessment and treatment of critically ill patients. Although the ABCDE approach is widely recommended, adherence in practice appears to be suboptimal. The cause of this non-compliance is unknown. As knowledge is a prerequisite for adherence, the aim of this study was to assess healthcare professionals’ knowledge of the ABCDE approach.

**Methods:**

A cross-sectional study was conducted at the Radboud University Medical Center, the Netherlands. A digital multiple-choice assessment tool of the ABCDE approach was developed by an expert panel through a mini-Delphi method and validated by performing test item statistics and an expert-novice comparison. The validated test was sent to healthcare professionals (nurses, residents and medical specialists) of the participating departments: Anaesthesiology, Paediatrics, Emergency Department and the Neonatal, Paediatric and Adult Intensive Care Units. Primary outcome was the test score, reflecting individual level of knowledge. Descriptive statistics, regression analysis and ANOVA were used.

**Results:**

Test validation showed a Cronbach’s alpha of 0.71 and an expert-novice comparison of 91.9% (standard deviation (SD) 9.1) and 72.4% (15.2) respectively (*p* < 0.001). Of 954 eligible participants, 240 filled out the questionnaire. The mean (SD) test score (% of correct answers) was 80.1% (12.2). Nurses had significantly lower scores (74.9% (10.9)) than residents (92.3% (7.5)) and medical specialists (88.0% (8.6)) (*p* < 0.001). The Neonatal Intensive Care Unit (75.9% (12.6)) and Adult Intensive Care Unit (77.4% (11.2)) had significantly lower scores than Paediatric Intensive Care Unit (85.6% (10.6)), Emergency Department (85.5% (10.4)) and Anaesthesiology (85.3% (10.6)) (*p* < 0.05). Younger participants scored higher than older participants (−0.30% (-0.46;-0.15) in test score/year increase in age).

**Conclusion:**

Scores of a validated knowledge test regarding the ABCDE approach vary among healthcare professionals caring for critically ill patients. Type of department, profession category and age had a significant influence on the test score. Further research should relate theoretical knowledge level to clinical practice. Tailored interventions to increase ABCDE-related knowledge are recommended.

**Supplementary Information:**

The online version contains supplementary material available at 10.1186/s12873-022-00753-y.

## Background

The Airway, Breathing, Circulation, Disability and Exposure (ABCDE) approach is a widely accepted, expert-based approach for the initial assessment and treatment of critically ill patients in all age categories, regardless of the underlying cause. It enables healthcare professionals to systematically assess and treat possibly fatal conditions in order of priority. Experts believe proper use of the ABCDE approach increases the quality of care and the approach is recommended in international guidelines and courses [1–4].

Despite efforts to increase awareness and knowledge of the ABCDE approach, adherence appears to be suboptimal [5, 6]. According to Olgers et al., healthcare professionals in the Emergency Department used the ABCDE approach in only 33% of potentially unstable patients [5]. A randomized controlled simulation study by Linders et al. found an overall adherence to the ABCDE approach performed by neonatal healthcare professionals of 31.5% [6]. The cause of this non-compliance is unknown. Many factors are known to thwart adherence in general. Algorithms may for example be unclear and it seems difficult to transfer knowledge and skills learned during courses to daily practice [7–10].

Based on experts’ belief that the ABCDE approach contributes to better patient care, and literature that strengthens this belief, it seems prudent to strive for optimal adherence to this algorithm [11, 12]. Knowing the reasons underlying non-adherence is essential to create tailored interventions. The aim of this study was to assess theoretical knowledge of healthcare professionals regarding the elements of the ABCDE approach.

## Methods

### Study design, study period and setting

A cross-sectional study was conducted at the Radboud University Medical Center in Nijmegen, the Netherlands, a university hospital with a bed capacity of approximately 1000 and over 11.000 employees. The participating departments were Anaesthesiology, Paediatrics, Emergency Department (ED), and the Neonatal (NICU), Paediatric (PICU) and Adult intensive care units (ICU), together employing 954 healthcare professionals. This study was conducted in 2019, of which the data collection took place from March 19th to April 28th, 2019. The Institutional Review Board of the Radboud University Medical Center considered our study exempt from formal approval, since participants were not exposed to medical interventions.

### Expert panel

An expert panel was assembled to develop an assessment tool on the ABCDE approach. The aim was to ensure that the knowledge assessment would be applicable to and relevant for all participants, and that it would cover all components of the approach. All participating departments were represented. The panel consisted of 2 neonatologists, 2 paediatricians, 2 anaesthesiologists, 1 critical care physician, 1 paediatric critical care physician and 1 emergency medicine physician. Furthermore, an educational specialist, an expert in test development, and an external expert on the ABCDE approach, were part of the expert panel.

### Test development

**Fig. 1:**
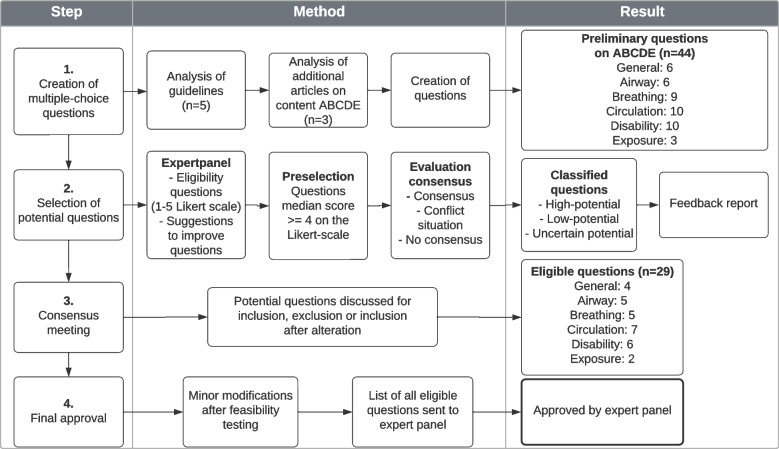
Development of the assessment tool

As no validated assessment tool applicable to all participants was available, one was developed using multiple-choice questions (MCQ) on the ABCDE approach. A 4-step mini modified Delphi consensus method was used (Fig. 1) [13]. First, guidelines and publications on the primary survey including the ABCDE approach were reviewed and preliminary MCQ were created [4, 14–20]. The expert panel rated these questions on a Likert scale from 1 (highly inapplicable) to 5 (highly applicable) and provided written feedback. The questions were edited based on the input of the panel members and evaluated in two steps: a preselection of questions with a median score ≥ 4 followed by evaluation of the degree of consensus. Consensus was considered acceptable if ≥70% scored 4 or 5 for a particular question. A ‘conflict situation’ arose if ≥30% scored 1 or 2 and ≥ 30% scored 4 or 5 for certain questions. In all other cases there was no consensus. Based on these steps, the questions were classified as high-potential (positive preselection and acceptable consensus), low-potential (negative preselection and no consensus) and uncertain-potential (all other combinations), similar to other studies [13, 21]. The expert panel received a report with the rating results and adjusted questions. This report was discussed in a consensus meeting and the questions were either accepted, rejected, or accepted after adjustment. This resulted in a final draft of the assessment tool, which underwent feasibility testing. Seven healthcare professionals from different departments (3 NICU, 3 PICU, 1 Anaesthesiology) and professions (2 nurses, 3 residents, 2 medical specialists) were requested to provide feedback on the readability, comprehensibility and relevance of the questions, as well as the time required to complete the test. Based on their feedback, minor modifications were made, after which the final version was sent to the expert panel for approval. This final version contained 29 multiple-choice questions (Additional file 1).

### Test validation

For the validation process, the validity framework of Cook et al. was used [22, 23]. Content validity was ensured, since all items were derived from manuals and guidelines, consensus on the test was reached among experts and a feasibility test was performed. To validate the test regarding internal structure and relationship with other variables, test item statistics and expert-novice comparison were performed. Item statistics was performed on the test results of the healthcare professionals and discussed with an expert on test development. A Cronbach’s alpha of > 0.60 was considered acceptable, since the knowledge test was used as a formative assessment [24, 25]. The reference values per item were a p’-value (proportion of participants that answered the question correctly) of 0.1–0.9 and Rir-value (distinctiveness of a question) of > 0.10 [26]. The Rir-value is the item-rest correlation: the correlation between an item score and the total score of all remaining items of the test [26, 27]. For the expert-novice comparison, the knowledge test was sent to life support course instructors (experts) and medical students ranging from first to sixth-year (novices), after which the test scores were compared.

### Selection of participants

Convenience sampling was used. All nurses, nurse practitioners (NP), physician assistants (PA), residents and medical specialists, employed at the participating departments, were approached to participate. The eventual population was formed by the healthcare professionals that filled out the questionnaire. Awareness of the ABCDE approach among the eligible participants was presumed since it is an universally applied algorithm, it is part of the education of all healthcare professionals in the Netherlands and the participating departments had exposure to critically ill patients. Healthcare professionals without clinical exposure to critically ill patients were excluded.

### Data collection

All eligible participants were informed about the study by the expert panel members, and approached for participation by email from March 19th to April 28th, 2019. This email contained a personal link to the questionnaire, which was made in Castor EDC, version 2019.1.8 (Castor, Amsterdam, the Netherlands). The questionnaire consisted of two parts. The first part surveyed demographic and clinical characteristics (Table 1). The second part contained the assessment tool on the contents of the ABCDE approach (Additional file 1). The questionnaire could be filled out at any time during a 4-week period. Participation was voluntary and could be withdrawn at any time. Participants were instructed not to study for the test, nor to use external information sources during the test. The progress was monitored with Castor EDC. Participants received a reminder after 1.5 and 3 weeks. Participants were classified as non-responders if they failed to complete the questionnaire before the deadline. The Institutional Review Board of the Radboud University Medical Center considered the study exempt from formal approval (file number CMO: 2018–4993). Informed consent was obtained from all participants. Data were anonymously stored on a secured server.

**Table 1:** Demographic and clinical characteristics of the participants (*n* = 240)

Total participants (n)	240		
Female (n)	178 (74%)		
Median age (years)	37 (IQR 31–52)		
Median working experience (years)	14 (IQR 7–27)		
	**n**	**/N**	**%**
**Profession category**			
Nurse	149	/240	62%
Nurse practitioner / Physician assistant	9	/240	4%
Resident	37	/240	15%
Medical specialist	45	/240	19%
*Profession category per department*			
**Department of Anaesthesiology**	**31**	**/240**	**13%**
Nurses	3	/31	10%
NP / PA	3	/31	10%
Residents	10	/31	32%
Medical specialists	15	/31	48%
**Department of Paediatrics**	**38**	**/240**	**16%**
Nurses	26	/38	68%
NP / PA	1	/38	3%
Residents	4	/38	11%
Medical specialists	7	/38	18%
**Emergency Department**	**23**	**/240**	**9%**
Nurses	15	/23	65%
NP / PA	0	/23	0%
Residents	7	/23	30%
Medical specialists	1	/23	5%
**Intensive Care Unit**	**80**	**/240**	**33%**
Nurses	63	/80	79%
NP / PA	1	/80	1%
Residents	8	/80	10%
Medical specialists	8	/80	10%
**Neonatal Intensive Care Unit**	**35**	**/240**	**15%**
Nurses	23	/35	65%
NP / PA	3	/35	9%
Residents	3	/35	9%
Medical specialists	6	/35	17%
**Paediatric Intensive Care Unit**	**33**	**/240**	**14%**
Nurses	19	/33	58%
NP / PA	1	/33	3%
Residents	5	/33	15%
Medical specialists	8	/33	24%
**Median interval since last education (months)**	8 (IQR 3–14)		
Nurses (*n* = 128)	8 (IQR 3–12)		
NP / PA (*n* = 8)	13 (IQR 4–20)		
Residents (*n* = 36)	6 (IQR 2–12)		
Medical specialists (*n* = 43)	12 (IQR 2–24)		
Missing (*n* = 25)			
**Previously attended life support courses (more than 1 possible) (n (%))**			
No life support education	24 (10%)		
Advanced Paediatric Life Support	65 (27%)		
Paediatric Advanced Life Support	29 (12%)		
European Paediatric Advanced Life Support	15 (6%)		
Advanced Cardiac Life Support	42 (18%)		
Advanced Trauma Life Support	48 (20%)		
Pre-Hospital Paediatric Life Support	7 (3%)		
Training by department	145 (60%)		
Theoretical lecture	76 (32%)		
Other life support education	47 (20%)		

*IQR* interquartile range, *NP* nurse practitioner, *PA* physician assistant

### Outcomes

The primary outcome was the individual test score (% of correct answers), reflecting the level of knowledge regarding the contents of the ABCDE approach. As secondary outcomes, factors that may have a bearing on knowledge were analysed. These included profession category, department of employment, age, gender, work experience and frequency and mode of training.

### Statistical analysis

Although this was an exploratory study, sample size calculation for multiple linear regression analysis with alpha 0.05, power 0.80, number of predictors *n* = 4 and estimated medium effect size of 0.13 revealed a sample size of *n* = 97 [28, 29]. Statistical analysis was performed using IBM SPSS statistics, version 25 (IBM Corporation, New York, USA). Depending on the probability distribution of the primary outcome, mean test score and standard deviation (SD) or median test score and interquartile range (IQR) were calculated. To evaluate the secondary outcomes, analyses-of-variance (ANOVA) with Tukey post hoc test and backward elimination regression analyses (using *p* > 0.2 to remove variables) were performed. A *p* < 0.05 was considered statistically significant for all tests.

## Results

### Validation

Item statistics showed a Cronbach’s alpha of 0.71. Eighteen of the 29 items had p’- and rir-values within the reference range. Eight items showed a p’-value > 0.9 (easy question) and five items a rir-value of < 0.1 (non-distinctive) (Additional file 2). Since the test was taken by healthcare professionals with an assumed prior level of knowledge, a higher p’-value for some items was expected and accepted. Deletion of items with a low rir-value did not improve the validity of the test. Adjusting items was not possible since the item analysis was performed after completion of the test. Therefore, no adjustments to the test were made.

A total of 72 participants, 48 instructors of life support courses and 23 medical students were included in the expert-novice comparison. The overall mean test score of the experts was 91.9% (SD 9.1%), compared to 72.4% (SD 15.2%) of the novices (*p* < 0.001).

### Knowledge test

All healthcare professionals of the participating departments (*n* = 954) were invited to participate, 243 filled out the questionnaire completely (response rate 25.5%). Three respondents were excluded for having no current exposure to patients. Of the remaining 240 participants, the majority were nurses (Table 1). The distribution of the profession categories and departments of the participants was comparable to the distribution of all approached healthcare providers.

The overall mean test score was 80.1% (SD 12.2). Test score differed per profession category and department (Table 2). The test score was significantly higher for residents and medical specialists compared to nurses (*p <* 0.001) and NP/PA (*p* < 0.05). Regarding the departments, the test score was significantly lower for participants from the NICU and ICU, compared to PICU, ED and Anaesthesiology (*p <* 0.05). There were no statistically significant differences between NICU and ICU or between the PICU, ED and Anaesthesiology (*p* > 0.90). The scores of the paediatric department were not significant different from any of the other departments.

**Table 2:** Test score per profession category and department

	Mean (SD) score (%)	95%-CI
**Overall (*n*** **= 240)**	80.1 (12.2)	78.6–81.7
Profession category*		
Nurse (*n* = 149) ^1 2^	74.9 (10.9)	73.2–76.7
Nurse practitioner / Physician assistant (*n* = 9) ^3 4^	78.1 (7.1)	73.5–82.7
Resident (*n* = 37) ^1 3^	92.3 (7.5)	89.9–94.7
Medical specialist (*n* = 45) ^2 4^	88.0 (8.6)	85.5–90.5
Department **		
Department of Anaesthesiology (*n* = 31) ^5 6^	85.3 (10.6)	81.6–89
Department of Paediatrics (*n* = 38)	77.8 (13.7)	73.4–82.2
Emergency department (*n* = 23) ^7 8^	85.5 (10.4)	81.3–89.8
Intensive care unit (*n* = 80) ^6 8 9^	77.4 (11.2)	75.0–80.0
Neonatal intensive care unit (*n* = 35) ^5 7 10^	75.9 (12.6)	71.7–80.1
Paediatric intensive care unit (*n* = 33) ^9 10^	85.6 (10.6)	82–89.2

* F(3,236) = 41.75, *p* < 0.001

** F(5, 234) = 5.818, p < 0.001

Differences in assessment scores of the ABCDE approach were analysed with one-way ANOVA. Identical superscript numbers indicate significant differences between groups in Tukey post hoc test (*p* < 0.05)

Backward elimination regression analyses yielded a model in which, besides department and profession category, age had a significant effect on the test score. Overall, younger participants scored higher on the knowledge test than older participants (−0.30% (0.46;-0.15) in test score for every year increase in age). No significant interactions were found between age, department and profession category. The only significant interaction was found between gender and profession category, since most nurses were female (87%) and most specialists were male (62%) (Additional file 3).

## Discussion

To our knowledge, this is the first study to explore theoretical knowledge specifically concerning the ABCDE approach. Healthcare professionals working with critically ill patients, scored on average 80% on a validated multiple-choice test involving the contents of the ABCDE approach. The type of department, profession category and age had a significant influence on the test score. Participants from the NICU and ICU scored lower than their colleagues from the PICU, Emergency Department and Anaesthesiology, residents and medical specialists outperformed nurses and NP/PA, and younger participants scored higher than more senior professionals. Apparently, there is variation in the level of knowledge of the ABCDE approach among different profession categories and various departments.

This study is the first to assess theoretical knowledge of the ABCDE approach among different disciplines and professions at a random moment. Previous studies did evaluate knowledge of the primary survey, but all in the context of life support courses and not regarding the ABCDE approach in particular [30–32]. Although comparison with previous research is difficult, all studies seem to support the importance of sufficient knowledge. Multiple studies corroborate that life support courses improve both theoretical knowledge and skills [30–32]. However, knowledge and skills deteriorate within 3–6 months without regular rehearsal [31, 33]. Since knowledge is a prerequisite for algorithm adherence, insufficiently acquired or retained knowledge may be a partial explanation for incomplete or incorrect application of the ABCDE approach [7, 10].

Although the ABCDE approach is well known and can be used in all patient categories, alternatives for or additions to the ABCDE approach have been created. An example of an alternative is the CAB approach, circulation-airway-breathing, in which the circulation is assessed first. This approach is important in cardiopulmonary resuscitation of patients with a cardiac arrest. Literature shows that this approach e.g. decreases the time to commencement of chest compressions [34]. However, the CAB approach is not recommended to use in the approach of critically ill patients without a cardiac arrest. An example of an addition is the paediatric assessment triangle (PAT) [35]. The PAT is a widely accepted tool for rapid, initial assessment of a child to establish the level of severity and to determine urgency for treatment. It precedes the ABCDE approach and does not replace it.

A study by Linders et al. assessed adherence to the approach during neonatal advanced life support scenarios. In line with the present study, lower adherence among nurses compared to residents and specialists was found [6]. Possible explanations are potential differences between the in-hospital courses for different profession categories or less accredited courses for nurses compared to physicians. Lastly, the amount of exposure could play a role, since more exposure facilitates retention of knowledge and skills [33, 36, 37]. Although all profession categories are expected to be familiar with the contents of the ABCDE approach, the resident usually performs the assessment when all profession categories are present.

This study shows that besides profession category, test scores differed between departments. One could argue this discrepancy could be partly attributed to the distribution of profession categories or discrepancy in age, but no significant interaction between these factors and department was present. The amount of exposure might play a role, although it is not likely to contribute considerably since all departments are caring for critically ill patients. Another possible explanation is that knowledge of the separate domains of the ABCDE approach might differ, related to the patient population. For example in Anaesthesiology, aspects of the airway might be more relevant or more frequently seen than neurological findings and the NICU does not have trauma patients. Although we tried to cover all parts of the ABCDE approach in the assessment tool, this might result in differences in test scores. Ongoing research focussing on adherence to the ABCDE approach in clinical practice might elucidate more on this subject.

The finding that younger participants scored higher on the test than the more senior participants was surprising. It can be assumed that more senior participants usually have more experience in clinical practice, have used the ABCDE approach more frequently and therefore score higher on the test. The fact that our results show otherwise, might be related to a difference in frequency, intensity and type of education or to a more executable role in clinical practice. Also, it is possible participants of a younger age are educated within a culture wherein the ABCDE approach is more universally acknowledged, but our data did not permit analysis of differences in education. At last, test score on theoretical knowledge cannot directly be related to adherence in clinical practice until proven otherwise.

Setting a cut-off score for passing or failing the test was difficult, since the optimal level of knowledge of the ABCDE approach cannot easily be determined [38–40]. It is unknown how the level of knowledge relates to clinical performance. Therefore it was used as a formative assessment tool without a threshold. However, based on the variation of the test score and the large standard deviations, knowledge of the ABCDE approach appears suboptimal in various healthcare professionals caring for critically ill patients.

### Strengths and limitations

The multidisciplinary approach of this study, makes it fairly unique. It gives a general insight in the level of knowledge of the ABCDE approach of healthcare professionals of various profession categories and departments. Furthermore, it is the first study that has specifically assessed theoretical knowledge of the ABCDE approach at a random moment, instead of in the context of life support courses, providing a more realistic view of the situation in clinical practice. The assessment tool was constructed using an evidence-based method for reaching consensus, with representatives from every department, an external expert on the subject, an educationalist and an expert on test development. The assessment tool was tested on feasibility by healthcare professionals of multiple participating professions and departments, so can be considered applicable to every participant. Lastly, the knowledge test was validated on multiple sources of validity evidence including test-item statistics and expert-novice comparison.

Some limitations arose while conducting this study. First, this was a single-centre study. Although the results of this study might not necessarily be completely generalizable, we still think the results can be applicable to healthcare professionals working in the same departments in similar hospitals in countries with a comparable healthcare system. Furthermore, the knowledge test that was developed, validated and used for this study can be used by researchers, educationalists or other people with interest in this topic. Second, the expert panel consisted of only medical specialists. Since the expert panel developed the assessment tool, the other profession categories might theoretically be disadvantaged. However, some members of the expert panel are instructors of courses for nurses and the MCQ was tested by a variety of participants of all profession categories, including nurses. Third, the questionnaire could be filled out at any time, without supervision, since a controlled setting could unfortunately not be created due to logistical reasons. Although participants were asked to abstain from seeking information sources and it was emphasized that the test score was processed anonymously and without individual consequences, this theoretically gave participants the opportunity to study, look up the answers to questions, or ask help of colleagues. It is unknown to what extend this might have affected the results. We hypothesized that a potential effect would lead to an increase in test scores, supporting the conclusion that test scores can be improved. Fourth, participants had attended different types of accredited and unaccredited life support courses, making it not feasible to differentiate between individual types of training.

Lastly, the overall response rate of 25.5% can be considered as limitation. A meta-analysis estimated the average survey response rate among healthcare professionals at 53%, although response rates < 30% are not uncommon [41–44]. In this study, the ratio of profession categories and departments of the participants is comparable to the ratio of all approached healthcare providers. Therefore, the results seem to be an adequate reflection of reality, although the response rate could affect the generalizability of the results. Possible explanations of the lower response rate are the length of the questionnaire, lack of interest in the subject matter, insufficient time, or the fact that it was a ‘test’. Although the number of non-responders and the probability of nonresponse bias are very poorly related, nonresponse bias cannot be excluded [45]. However, if only the most motivated healthcare professionals participated, it is likely that the average test score would otherwise have been lower, indicating an even greater need for education.

## Conclusions

In this study a validated assessment tool was developed and used, showing that theoretical knowledge of the contents of the ABCDE approach varies among healthcare professionals caring for critically ill patients. It could be hypothesized that sufficient knowledge facilitates consistent use of the ABCDE approach and therefore improves the quality of patient care. However, it is unknown which level of knowledge will improve clinical performance. The next step for further research is to relate theoretical knowledge to clinical practice and to uncover other factors influencing adherence to the ABCDE approach in practice.

## Supplementary Information

**Additional file 1.** Knowledge test (translated from Dutch).

**Additional file 2.** Item analysis.

**Additional file 3.** Regression analysis.

## Data Availability

The datasets used and analysed during the current study are available from the corresponding author on reasonable request.

## Abbreviations

ABCDE: Airway, breathing, circulation, disability, exposure
ED: Emergency department
MCQ: Multiple-choice questions
ICU: Adult intensive care unit
NICU: Neonatal intensive care unit
NP: Nurse practitioner
PA: Physician assistant
PICU: Paediatric intensive care unit
