# Supplementary material for: Healthcare professionals’ knowledge of the systematic ABCDE approach: a cross-sectional study
Source: BMC Emerg Med. 2022 Dec 12;22:202. doi: 10.1186/s12873-022-00753-y (PMC9743501; doi:10.1186/s12873-022-00753-y)
Supplement: Supplementary file 1 — Additional file 1. Knowledge test (translated from Dutch). [file 12873_2022_753_MOESM1_ESM.docx]

**Additional file 1**. Knowledge test (translated from Dutch)

Note: The questions below concern the assessment of a (potentially) critically ill patient with use of the ABCDE approach as part of the ‘Primary Survey’. It does not concern cardiopulmonary resuscitation.

The answer in *italics* is correct. Questions were asked in a random order

1. **What is usually a late sign of circulatory failure?**
   1. Hypertension
   2. *Hypotension*
   3. Hyperthermia
   4. Hypothermia
2. **Where is the airway obstruction located when you hear expiratory wheezing?**
   1. Upper airway
   2. *Lower airway*
   3. Expiratory wheezing does not provide information on the location of an obstruction
3. **At which locations can an internal bleeding cause a hypovolemic shock?**
   1. *Thorax, abdomen, pelvis, upper leg*
   2. Abdomen, upper arm, pelvis, upper leg
   3. Head, abdomen, upper arm, upper leg
4. **Which of the following aspects helps with assessing the effort of breathing, additional to frequency of breathing?**
   1. Cyanosis
   2. *Use of accessory breathing muscles*
   3. Oxygen saturation
5. **What blood value is always part of the primary survey?**
   1. Blood gas analysis
   2. *Glucose*
   3. Lactate
   4. Complete blood count
6. **A patient has a capillary refill time of 4 seconds on the sternum. This is...**
   1. *Abnormal*
   2. Normal
7. **On which of the following moments in the assessment of the patient do you use the ABCDE approach?**
   1. Initial assessment
   2. First reassessment
   3. *Both of the above*
   4. None of the above
8. **What does the P in the AVPU-score mean?**
   1. Patient responds to talking
   2. *Patient responds to pain*
   3. Patient does not respond to talking
   4. Patient does not respond to pain
9. **Classify the items below in order of priority according to the ABCDE approach:
   1. AVPU-score
   2. Assessing capillary refill time
   3. Look-listen-feel**
   1. 1, 2, 3
   2. 2, 3, 1
   3. *3, 2, 1*
   4. 1, 3, 2
10. **What aspect is not part of the D in the ABCDE approach?**
    1. AVPU-score
    2. Blood glucose level
    3. Pupil assessment
    4. *Temperature measurement*
11. **What is, at any age, most informative for determining an open airway?**
    1. No cyanosis
    2. No visible obstruction
    3. *Normal sound of voice (crying, talking)*
    4. Normal thorax movement
12. **Which of the following actions does not contribute to diagnosing a tension pneumothorax?**
    1. Auscultation
    2. Looking at jugular veins
    3. *Observing retractions*
    4. Percussion
13. **Which C-problem is treated prior to the actual ABCDE approach?**
    1. Anaphylactic shock
    2. Hypotension
    3. *Massive, visual haemorrhage*
    4. Potential cervical spine injury
14. **What are the 4 most essential parts for assessing a patients circulatory condition?**
    1. Auscultation heart, blood pressure, capillary refill time, pulsations
    2. Auscultation heart, blood pressure, capillary refill time, heart rate
    3. Blood pressure, capillary refill time, ECG, pulsations
    4. *Blood pressure, capillary refill time, heart rate, pulsations*
15. **Which of the following aspects is assessed in the E of the ABCDE approach?**
    1. Cyanosis
    2. Cold extremities
    3. *Petechiae*
16. **Which of the following parameters is most reliable when assessing the effectiveness of breathing (in ambient air)?**
    1. Frequency of breathing
    2. Cyanosis
    3. Nose flaring
    4. *Oxygen saturation*
17. **What is the most uniform sign of increased effort of breathing, for all ages?**
    1. Use of accessory breathing muscles
    2. Retractions
    3. *Increased breathing rate*
    4. Decreased oxygen saturation
18. **Upon what is the fixed order of the ABCDE approach mostly based?**
    1. This is based upon results of randomized research
    2. This is based upon practicability
    3. *This is based upon priorit*y
19. **Which item is assessed in the E of the ABCDE approach?**
    1. Blood glucose
    2. *Body temperature*
    3. Instability of the cervical spine
    4. Blood pressure

1. **What is not a sign of shock?**
   1. Cold extremities
   2. *Polyuria*
   3. Prolonged capillary refill time
   4. Weak pulsations
2. **A prolonged capillary refill time could be present in which of the following situations?**1. Hypertension
   2. Hypothermia
   3. Shock
   1. 1 and 2
   2. 1 and 3
   3. *2 and 3*
3. **Which abnormality in a patient’s posture is most severe?**
   1. Single sided, rhythmic movement of an arm
   2. Single sided weakness of an arm
   3. Rhythmic movement of arms and legs
   4. *Stretching arms and legs*
4. **What is the safest manoeuvre to secure the airway of a trauma patient?**
   1. Head-tilt, combined with chin lift
   2. Head-tilt
   3. Head-tilt, combined with jaw thrust
   4. *Jaw thrust*
5. **Which aspects of the pupils always have to be assessed for every critically ill patient?**
   1. *Size and light reactivity*
   2. Size and cloudiness
   3. Light reactivity and shape
   4. Shape and cloudiness

1. **What is the look-listen-feel method namely meant for?**
   1. Determining a change in awareness
   2. *Assessing the airway*
   3. Determining the frequency of breathing
   4. Diagnosing a pneumothorax
2. **Which muscle is an accessory breathing muscle?**
   1. Diaphragm
   2. Intercostal muscle
   3. *Sternocleidomastoid muscle*
3. **What does a stridor (inspiratory) indicate?**
   1. Severe asthma attack
   2. *Upper airway obstruction*
   3. Lower airway obstruction
   4. Airway obstruction (regardless of level)
4. **What does jugular venous distension indicate?
   1. Anaphylaxis
   2. Heart failure
   3. Pericardial tamponade
   4. Pneumothorax**
   1. 1, 2, 3
   2. 1, 2, 4
   3. 1, 3, 4
   4. *2, 3, 4*
5. **With what can you make a quick estimation of a patients awareness?**
   1. *AVPU-score*
   2. Glasgow Coma Scale
   3. EEG
   4. Pupil examination
