# Supplementary material for: Healthcare professionals’ knowledge of the systematic ABCDE approach: a cross-sectional study
Source: BMC Emerg Med. 2022 Dec 12;22:202. doi: 10.1186/s12873-022-00753-y (PMC9743501; doi:10.1186/s12873-022-00753-y)
Supplement: Supplementary file 2 — Additional file 2. Item analysis. [file 12873_2022_753_MOESM2_ESM.pdf]

Additional file 2. Regression analysis

Knowledge test ABCDE approach

PROBABILITY SCORE **8,08**

Question	Correct answer		Maximum score achieved	
	mean	stand.dev.	lowest	highest
Testscore	23,24	3,54	13,00	29,00
Correct	23,24	3,53	13,00	29,00
Incorrect	5,76	3,53	16,00	0,00
Open	0,00	0,00	0	0

ITEMANALYSIS SUMMARY

	mean	stand.dev.	lowest	highest
Proportion correct	0,80	0,16	0,40	1,00
Idem corrected	0,73	0,22	0,10	1,00
Item-total correlation	0,32	0,14	0,09	0,58
Item-rest correlation	0,23	0,13	0,02	0,48

coefficient alpha:	0,71
standard error:	1,90

TEST CHARACTERISTICS

Total number of items	29
Number of items excluded	0
Eventual number of items	29
Number of 2-choice items:	1
Number of 3-choice items:	7
Number of 4-choice items:	21
Number of 5-choice items:	0
Number of alternatives	107
Number of participants	240

Additional file 2. Regression analysis

ITEMANALYSIS

Question	Correct answer	Maximum possible score	Maximum score achieved	Minimum score achieved	Mean score	Stand. Dev.	p	p"	Rit	Rir	open		1		2		3		4	
											f	z	f	z	f	z	f	z	f	z
1	1	1	1	0,00	0,72	0,45	0,72	0,62	0,35	0,23	0	*	172	0,14	6	-0,89	3	-1,92	59	-0,23
2	1	1	1	0,00	0,77	0,42	0,77	0,66	0,41	0,31	0	*	185	0,17	26	-1,07	29	-0,10		
3	1	1	1	0,00	0,93	0,25	0,93	0,90	0,24	0,17	0	*	224	0,04	10	-0,29	6	-1,19		
4	1	1	1	1,00	1,00	0,00	1,00	1,00	*	*	0	*	240	0,00	0	*	0	*		
5	1	1	1	0,00	0,53	0,50	0,53	0,38	0,56	0,45	0	*	128	0,42	89	-0,44	13	-0,54	10	-0,76
6	1	1	1	0,00	0,95	0,21	0,95	0,91	0,17	0,11	0	*	229	0,02	11	-0,50				
7	1	1	1	0,00	0,93	0,25	0,93	0,91	0,09	0,02	0	*	224	0,00	13	0,17	2	-1,22	1	-0,94
8	1	1	1	0,00	0,95	0,21	0,95	0,94	0,20	0,14	0	*	229	0,03	3	0,01	1	-2,65	7	-0,65
9	1	1	1	0,00	0,72	0,45	0,72	0,62	0,30	0,18	0	*	172	0,11	8	-0,41	2	-0,74	58	-0,25
10	1	1	1	0,00	0,81	0,39	0,81	0,74	0,47	0,38	0	*	194	0,19	12	-0,45	24	-0,96	10	-0,75
11	1	1	1	0,00	0,91	0,29	0,91	0,88	0,31	0,24	0	*	218	0,07	0	*	5	-1,43	17	-0,54
12	1	1	1	0,00	0,48	0,50	0,48	0,31	0,58	0,48	0	*	116	0,49	5	-0,54	38	-0,71	81	-0,33
13	1	1	1	0,00	0,85	0,36	0,85	0,79	0,27	0,17	0	*	203	0,07	11	-0,80	2	-0,40	24	-0,21
14	1	1	1	0,00	0,87	0,34	0,87	0,82	0,30	0,21	0	*	208	0,08	9	-0,08	11	-1,03	12	-0,42
15	1	1	1	0,00	0,91	0,28	0,91	0,87	0,37	0,30	0	*	219	0,09	0	*	21	-0,97		
16	1	1	1	0,00	0,78	0,41	0,78	0,71	0,17	0,05	0	*	188	0,03	36	-0,13	9	0,35	7	-0,54
17	1	1	1	0,00	0,43	0,50	0,43	0,24	0,26	0,12	0	*	104	0,14	120	-0,04	13	-0,37	3	-1,40
18	1	1	1	0,00	0,97	0,18	0,97	0,95	0,13	0,08	0	*	232	0,01	4	-0,86	4	-0,01		
19	1	1	1	0,00	0,70	0,46	0,70	0,59	0,56	0,46	0	*	167	0,30	46	-0,86	27	-0,41	0	*
20	1	1	1	0,00	0,93	0,26	0,93	0,90	0,28	0,20	0	*	222	0,06	8	-0,85	2	-1,39	8	-0,42
21	1	1	1	0,00	0,97	0,17	0,97	0,96	0,15	0,10	0	*	233	0,02	1	1,35	6	-0,88		
22	1	1	1	0,00	0,83	0,38	0,83	0,77	0,32	0,22	0	*	199	0,10	0	*	24	-0,23	17	-0,84
23	1	1	1	0,00	0,79	0,41	0,79	0,72	0,42	0,31	0	*	189	0,16	29	-0,64	0	*	22	-0,55
24	1	1	1	0,00	0,85	0,36	0,85	0,79	0,26	0,17	0	*	203	0,07	1	-0,98	36	-0,37	0	*
25	1	1	1	0,00	0,83	0,37	0,83	0,78	0,18	0,07	0	*	200	0,03	13	-0,65	17	0,25	10	-0,23
26	1	1	1	0,00	0,40	0,49	0,40	0,10	0,46	0,34	0	*	96	0,42	20	-0,63	124	-0,22		
27	1	1	1	0,00	0,75	0,43	0,75	0,67	0,41	0,30	0	*	181	0,17	2	0,15	11	-0,84	46	-0,47
28	1	1	1	0,00	0,85	0,36	0,85	0,79	0,49	0,41	0	*	203	0,17	15	-1,09	7	-0,80	15	-0,89
29	1	1	1	0,00	0,83	0,37	0,83	0,78	0,39	0,30	0	*	200	0,13	37	-0,62	0	*	3	-1,20
