# Supplementary material for: Healthcare professionals’ knowledge of the systematic ABCDE approach: a cross-sectional study
Source: BMC Emerg Med. 2022 Dec 12;22:202. doi: 10.1186/s12873-022-00753-y (PMC9743501; doi:10.1186/s12873-022-00753-y)
Supplement: Supplementary file 3 — Additional file 3. Regression analysis. [file 12873_2022_753_MOESM3_ESM.docx]

**Additional file 3.** Regression analysis

| **Tests of Between-Subjects Effects** | | | | | | | | | | |  |
| --- | --- | --- | --- | --- | --- | --- | --- | --- | --- | --- | --- |
| Dependent Variable: Test score (%) | | | | | | | | | | |  |
| Source | Type III Sum of Squares | | df | | Mean Square | | F | | P-value | |  |
| Corrected Model | 15546,256^a^ | | 22 | | 706,648 | | 7,944 | | ,000 | |  |
| Intercept | 69063,729 | | 1 | | 69063,729 | | 776,381 | | ,000 | |  |
| Sex | 164,274 | | 1 | | 164,274 | | 1,847 | | ,176 | |  |
| Department | 1058,762 | | 5 | | 211,752 | | 2,380 | | ,040 | |  |
| Profession category | 2759,295 | | 2 | | 1379,647 | | 15,509 | | ,000 | |  |
| Age | 516,029 | | 1 | | 516,029 | | 5,801 | | ,017 | |  |
| Interaction sex * age | 173,871 | | 1 | | 173,871 | | 1,955 | | ,164 | |  |
| Interaction department * profession category | 1347,098 | | 10 | | 134,710 | | 1,514 | | ,137 | |  |
| Interaction sex * profession category | 846,208 | | 2 | | 423,104 | | 4,756 | | ,010 | |  |
| Error | 16634,765 | | 187 | | 88,956 | |  | |  | |  |
| Total | 1413400,713 | | 210 | |  | |  | |  | |  |
| Corrected Total | 32181,020 | | 209 | |  | |  | |  | |  |
| a. R Squared = ,483 (Adjusted R Squared = ,422) | | | | | | | | | | |  |
| **Parameter Estimates** | | | | | | | | | | | |
| Dependent Variable: Test score (%) | | | | | | | | | | | |
| Parameter | B | Std. Error | | t | | P-value | | 95% Confidence Interval | | | |
|  |  |  |  |  |  |  |  | Lower Bound | | Upper Bound | |
| Intercept | 102,349 | 4,872 | | 21,006 | | ,000 | | 92,737 | | 111,961 | |
| Male | -15,694 | 8,402 | | -1,868 | | ,063 | | -32,270 | | ,882 | |
| Female | 0^a^ | . | | . | | . | | . | | . | |
| NICU | 3,082 | 5,929 | | ,520 | | ,604 | | -8,614 | | 14,779 | |
| PICU | 10,001 | 5,143 | | 1,944 | | ,053 | | -,146 | | 20,147 | |
| ICU | 2,699 | 5,360 | | ,504 | | ,615 | | -7,875 | | 13,273 | |
| Emergency Department | 9,388 | 10,714 | | ,876 | | ,382 | | -11,748 | | 30,523 | |
| Department of Anaesthesiology | -,465 | 4,698 | | -,099 | | ,921 | | -9,732 | | 8,802 | |
| Department of Paediatrics | 0^a^ | . | | . | | . | | . | | . | |
| Nurses | -20,232 | 4,460 | | -4,536 | | ,000 | | -29,032 | | -11,433 | |
| NP/PA/Residents | 4,327 | 6,147 | | ,704 | | ,482 | | -7,799 | | 16,453 | |
| Medical specialists | 0^a^ | . | | . | | . | | . | | . | |
| Age | -,302 | ,077 | | -3,902 | | ,000 | | -,455 | | -,150 | |
| Interaction Male * Age | ,221 | ,158 | | 1,398 | | ,164 | | -,091 | | ,532 | |
| Interaction Female * Age | 0^a^ | . | | . | | . | | . | | . | |
| Interaction NICU * Nurses | ,640 | 6,579 | | ,097 | | ,923 | | -12,339 | | 13,618 | |
| Interaction NICU * NP/PA/Residents | -17,704 | 8,291 | | -2,135 | | ,034 | | -34,060 | | -1,348 | |
| Interaction NICU * Medical specialists | 0^a^ | . | | . | | . | | . | | . | |
| Interaction PICU * Nurses | ,343 | 5,949 | | ,058 | | ,954 | | -11,393 | | 12,080 | |
| Interaction PICU * NP/PA/Residents | -15,146 | 7,838 | | -1,933 | | ,055 | | -30,608 | | ,315 | |
| Interaction PICU * Medical specialists | 0^a^ | . | | . | | . | | . | | . | |
| Interaction ICU * Nurses | 1,373 | 5,880 | | ,234 | | ,816 | | -10,226 | | 12,973 | |
| Interaction ICU * NP/PA/Residents | -12,196 | 7,665 | | -1,591 | | ,113 | | -27,317 | | 2,924 | |
| Interaction ICU * Medical specialists | 0^a^ | . | | . | | . | | . | | . | |
| Interaction Emergency Department * Nurses | 1,166 | 11,273 | | ,103 | | ,918 | | -21,073 | | 23,406 | |
| Interaction Emergency Department * NP/PA/Residents | -14,048 | 12,156 | | -1,156 | | ,249 | | -38,028 | | 9,933 | |
| Interaction Emergency Department * Medical Specialists | 0^a^ | . | | . | | . | | . | | . | |
| Interaction Department of Anaesthesiology * Nurses | -3,220 | 8,392 | | -,384 | | ,702 | | -19,775 | | 13,334 | |
| Interaction Department of Anaesthesiology * NP/PA/Residents | -2,658 | 6,967 | | -,381 | | ,703 | | -16,401 | | 11,086 | |
| Interaction Department of Anaesthesiology * Medical Specialsists | 0^a^ | . | | . | | . | | . | | . | |
| Interaction Department of Paediatrics * Nurses | 0^a^ | . | | . | | . | | . | | . | |
| Interaction Department of Paediatrics * NP/PA/Residents | 0^a^ | . | | . | | . | | . | | . | |
| Interaction Department of Paediatrics * Medical Specialists | 0^a^ | . | | . | | . | | . | | . | |
| Interaction Male * Nurses | 13,293 | 4,441 | | 2,993 | | ,003 | | 4,533 | | 22,053 | |
| Interaction Male * NP/PA/Residents | 6,180 | 5,108 | | 1,210 | | ,228 | | -3,896 | | 16,256 | |
| Interaction Male * Medical Specialists | 0^a^ | . | | . | | . | | . | | . | |
| Interaction Female * Nurses | 0^a^ | . | | . | | . | | . | | . | |
| Interaction Female * NP/PA/Residents | 0^a^ | . | | . | | . | | . | | . | |
| Interaction Female * Medical Specialists | 0^a^ | . | | . | | . | | . | | . | |
| a. This parameter is set to zero because it is redundant. | | | | | | | | | | | |
